# Transposable Element Insertions in Long Intergenic Non-Coding RNA Genes

**DOI:** 10.3389/fbioe.2015.00071

**Published:** 2015-06-09

**Authors:** Sivakumar Kannan, Diana Chernikova, Igor B. Rogozin, Eugenia Poliakov, David Managadze, Eugene V. Koonin, Luciano Milanesi

**Affiliations:** ^1^National Center for Biotechnology Information, National Library of Medicine, National Institutes of Health, Bethesda, MD, USA; ^2^Department of Genetics, Institute for Quantitative Biomedical Sciences, Geisel School of Medicine, Dartmouth College, Hanover, NH, USA; ^3^Laboratory of Retinal Cell and Molecular Biology, National Eye Institute, National Institutes of Health, Bethesda, MD, USA; ^4^Institute for Biomedical Technologies, National Research Council, Segrate, Italy

**Keywords:** mobile elements, molecular domestication, exaptation, junk DNA, long non-coding RNA, repetitive elements

## Abstract

Transposable elements (TEs) are abundant in mammalian genomes and appear to have contributed to the evolution of their hosts by providing novel regulatory or coding sequences. We analyzed different regions of long intergenic non-coding RNA (lincRNA) genes in human and mouse genomes to systematically assess the potential contribution of TEs to the evolution of the structure and regulation of expression of lincRNA genes. Introns of lincRNA genes contain the highest percentage of TE-derived sequences (TES), followed by exons and then promoter regions although the density of TEs is not significantly different between exons and promoters. Higher frequencies of ancient TEs in promoters and exons compared to introns implies that many lincRNA genes emerged before the split of primates and rodents. The content of TES in lincRNA genes is substantially higher than that in protein-coding genes, especially in exons and promoter regions. A significant positive correlation was detected between the content of TEs and evolutionary rate of lincRNAs indicating that inserted TEs are preferentially fixed in fast-evolving lincRNA genes. These results are consistent with the repeat insertion domains of LncRNAs hypothesis under which TEs have substantially contributed to the origin, evolution, and, in particular, fast functional diversification, of lincRNA genes.

## Introduction

Traditionally, genomes have been perceived mostly as repositories of protein-coding genes. Although this might be largely true in the case of viruses, prokaryotes, and unicellular eukaryotes, numerous recent studies on the genomes of multicellular eukaryotes, particularly animals, have revealed a vast non-coding RNome, i.e., numerous genes encoding various classes of non-coding RNAs (ncRNAs) (Carninci et al., 2005; Mattick and Makunin, 2006; Ponting et al., 2009; Derrien et al., 2012; Amaral et al., 2013). Strikingly, the total number of genes for ncRNAs that are expressed from a mammalian genome seems to exceed the number of protein-coding genes several fold (Mattick and Makunin, 2006; Amaral et al., 2013). The classification of ncRNAs and validation of their functionality remain matters of intensive investigation and debate (Van Bakel and Hughes, 2009; Ponting and Belgard, 2010; Graur et al., 2013). Among many distinct classes of ncRNAs, the long non-coding RNA (lncRNA) is probably the most enigmatic group. The definition of a lncRNA is based solely on the transcript size: lncRNAs are defined as ncRNAs longer than 200 nt (Mattick and Makunin, 2006; Ponting et al., 2009). Many lncRNAs are spliced, 5′capped, and polyadenylated (Okazaki et al., 2002; Carninci et al., 2005; Kapranov et al., 2007; Ponjavic et al., 2007). Based on the localization in the genome, lncRNAs can be divided into two distinct classes: (i) transcripts that overlap protein-coding genes, many of which are likely to be involved in sense–antisense regulation (Chen et al., 2005; Ponting and Belgard, 2010; Rinn and Chang, 2012) and (ii) long intergenic non-coding (linc)RNAs that are transcribed from genome regions separating protein-coding genes (Ponjavic et al., 2007; Mercer et al., 2008; Ponting et al., 2009).

The current knowledge on the functions of long intergenic non-coding RNAs (lincRNAs) is scarce because very few of the lincRNAs have been experimentally characterized. Nevertheless, the functional range of this class of ncRNA is believed to be broad on the basis of indirect evidence (Bertone et al., 2004; Ponjavic et al., 2007; Mercer et al., 2008; Ponting and Belgard, 2010; Ulitsky et al., 2011; Glazko et al., 2012; Ng et al., 2013). It has been proposed that lincRNAs could be involved in the regulation of many cellular processes (Mattick and Makunin, 2006; Loewer et al., 2010; Wang et al., 2011; Rinn and Chang, 2012). For example, they can affect transcription locally on the gene level (Martens et al., 2004; Martianov et al., 2007; Osato et al., 2007; Hirota et al., 2008) as well as target transcription regulators and thus affect transcription of many genes (Feng et al., 2006; Goodrich and Kugel, 2006). They can also target RNA polymerase II in human and mouse (Espinoza et al., 2007; Mariner et al., 2008) and thus affect the expression of an even broader range of genes. Furthermore, lincRNAs participate in the regulation of splicing (Munroe and Lazar, 1991; Beltran et al., 2008) and translation (Wang et al., 2005; Centonze et al., 2007). Well-characterized examples of lincRNAs involved in epigenetic processes are *Xist* (Brockdorff et al., 1992; Elisaphenko et al., 2008), *Kcnq1ot1* (Umlauf et al., 2004; Pandey et al., 2008), and *Air* (Nagano et al., 2008).

It is well established that, compared to protein-coding sequences and structural RNAs, lincRNAs are weakly conserved in evolution. Many early studies, therefore, branded the lincRNAs “transcriptional dark matter” and considered them to be generally non-functional (Van Bakel and Hughes, 2009; Robinson, 2010). However, low level or lack of detectable conservation does not necessarily imply that these molecules have no function (Pang et al., 2006). A case in point is the best-characterized, functionally important lincRNA gene, *Xist*, which is weakly conserved although it does contain evolutionary constrained regions (Elisaphenko et al., 2008). In general, lincRNAs show reduced substitution and insertion–deletion rates, which has been attributed to purifying selection (Ponjavic et al., 2007; Managadze et al., 2011). Taking into account that some lincRNA genes originated from protein-coding genes [for example, *Xist* (Duret et al., 2006; Elisaphenko et al., 2008)], it appears likely that many properties of lincRNAs would generally resemble those of protein-coding genes, despite the typically lower level of constraint. In particular, protein-coding genes that are highly expressed in many tissues typically evolve slower than genes with lower expression level and breadth (Duret and Mouchiroud, 2000; Krylov et al., 2003; Drummond and Wilke, 2008), and a similar dependence has been observed for lincRNA genes (Managadze et al., 2011). Taken together, these findings imply that an unknown but substantial fraction of lincRNAs are functional molecules rather than transcriptional noise and have evolutionary properties similar to those of protein-coding genes. However, the number of functionally characterized lincRNAs remains scarce (Amaral et al., 2013).

The origin of lincRNA genes generally remains enigmatic. However, analysis of the well-characterized *Xist* lincRNA has revealed fragmentary homology to a protein-coding gene *Lnx3* suggesting that the *Xist* genes emerged in early eutherians via integration of transposable elements (TEs) into the *Lnx3* gene, which gave rise to simple tandem repeats (Duret et al., 2006; Elisaphenko et al., 2008). The *Xist* gene promoter region and 4 of its 10 exons retain homology to exons of the *Lnx3* gene. The remaining six *Xist* exons including those containing simple tandem repeats show similarity to different TEs (Elisaphenko et al., 2008). Integration of TEs into the *Xist* gene apparently had been occurring throughout the course of evolution of this gene and most likely continues in contemporary eutherian species. Additionally, it has been shown that the combination of remnants of protein-coding sequences and TEs is not unique to the *Xist* gene but is also found in neighboring genes that encode non-coding nuclear RNAs (Elisaphenko et al., 2008; Kolesnikov and Elisafenko, 2010).

The discovery of the pivotal contribution of TEs to the evolution of the *Xist* gene prompts the question on a possible general role of TEs in the evolution of lincRNAs. Diverse TEs are widespread and abundant in the genomes of most eukaryotes (Smit, 1996; Brosius, 1999; Kidwell and Lisch, 2001; Deininger and Batzer, 2002). Different classes of TEs include mobile retrovirus-like elements, or retrotransposons, which transpose within the genome via RNA intermediates, and DNA transposons, which can relocate directly. Retrotransposons including long interspersed repetitive elements (LINEs), short interspersed repetitive elements (SINEs), and long terminal repeat (LTR) retrotransposons are widely represented in mammals (Smit, 1996; Deininger and Batzer, 2002). The LINEs are transcribed by RNA polymerase II and contain open reading frames (ORFs) (Temin, 1985). A complete and transpositionally active L1 element (the most common variety of LINEs) is ~7 kb long and contains a 5′-untranslated region (UTR) with an internal promoter, two ORFs (ORF1 and ORF2) and a 3′-UTR terminated by a polyadenylate-rich tail (Smit, 1996; Deininger and Batzer, 2002). The ORF1 encodes a putative RNA-binding protein ~40 kDa in size (Martin, 2006) whereas ORF2 encodes a protein with endonuclease and reverse transcriptase (RT) activities that generates cDNAs from RNA transcripts of the element (Loeb et al., 1986). The mobility of the LINE elements had been demonstrated in mouse and human genomes (Kazazian et al., 1988; Boccaccio et al., 1990). The SINEs are characterized by the presence of a split intragenic RNA polymerase III promoter and a 3′A-rich region often followed by an oligo(A) tail (Smit, 1996; Rogozin et al., 2000; Kapitonov and Jurka, 2003). The SINEs do not contain long ORFs and do not encode enzymes for transposition. Instead, transposition of SINEs apparently requires RT encoded by other TEs, in particular, LINEs (Smit, 1996; Deininger and Batzer, 2002). The LTR retrotransposons have LTRs that range from ~100 bp to over 5000 bp in size (Smit, 1996; Deininger and Batzer, 2002). The LTR retrotransposons are similar to retroviruses in organization, with transcriptional regulatory sequences located in the flanking LTRs, a RT priming site that is typically located immediately downstream of an first LTR, and several ORFs encoding proteins involved in retrotransposition, in particular, RT and integrase (Smit, 1996; Deininger and Batzer, 2002).

The TEs are the primary contributors to the bulk of the genomic DNA in many eukaryotes, in particular mammals, and have the potential to contribute to the evolution of the hosts by providing novel regulatory or coding sequences (Makalowski, 2000). Different classes of regulatory regions in the human genome have been surveyed for the presence of TE-derived sequences (TES) to systematically assess the potential contribution of TEs to the regulation of human genes, and almost 25% of the analyzed promoter regions have been found to contain TES (Jordan et al., 2003; Feschotte, 2008; Bourque, 2009). In addition, numerous examples where experimentally characterized *cis*-regulatory elements are derived from TE sequences have been identified (Jordan et al., 2003; Bourque et al., 2008; Faulkner et al., 2009). Thus, thousands of human (and other mammalian) genes appear to be regulated, at least in part, by sequences derived from TEs (Jordan et al., 2003; Feschotte, 2008; Bourque, 2009). The TES are likely to have substantially contributed to evolutionary change in both gene specific and global patterns of mammalian protein-coding gene regulation (Makalowski, 2000; Jordan et al., 2003).

In light of the regulatory and structural effects that some TEs exert on host protein-coding and lncRNA genes (Makalowski, 2000; Jordan et al., 2003; Elisaphenko et al., 2008; Mattick et al., 2010; Wang et al., 2011; Kapusta et al., 2013; Johnson and Guigo, 2014), we sought to examine the contribution and conservation of TES to regulatory regions, exons and introns of human and mouse lincRNA genes. We found that introns of lincRNA genes contain the highest fraction of TES, followed by exons. The promoters of the lincRNAs contain the lowest fraction of TES but the largest fraction of ancient TES that are conserved between primates and rodents. The content of TES in lincRNA genes is substantially greater than in protein-coding genes, particularly in exons and promoter regions. These results are compatible with the view that TEs are major contributors to the origin and evolution of lincRNAs. We further sought to assess the potential utility of TES as an “evolutionary variable” by analyzing the correlations between the TES content, lincRNA expression, and sequence conservation.

## Materials and Methods

Human and mouse lincRNA genes, the corresponding genomic alignments and expression data were taken from our previous work (Managadze et al., 2011) where the procedures of data processing are described in full details. Briefly, complete mouse and human probe sets were downloaded from the NRED database (Dinger et al., 2009) in the tab delimited and browser extensible data (BED, containing genomic coordinates) formats. The probe sets from platform GNF Atlas 2 (Mouse and Human), with the target classification “Non-coding Only,” were used for further analysis. This protocol yielded 917 human and 5444 mouse probe sets. Only the probe sets that mapped to intergenic regions of the human and mouse genomes (i.e., between two adjacent protein-coding genes) were used for further analysis. The resulting list of lincRNAs was further filtered: sequences shorter than 200 nt were removed. This procedure yielded the final set of NCBI GenBank Accession IDs of 2390 mouse and 589 human lincRNAs and their corresponding microarray expression probe sets. The genomic coordinates and sequences of exons and introns of lincRNA and protein-coding genes were downloaded from the UCSC Table Browser (Karolchik et al., 2004), specifically, from “all_mrna” tables of mouse mm8 and human hg18 assemblies. Multiple alignments of these regions were fetched from Galaxy (Goecks et al., 2010). For the detection of TES, lincRNA and protein-coding genes were analyzed using RepeatMasker version open-3.1.3^1^ with the following parameters: -w -s -no_is -cutoff 255 -frag 20000 -gff -species mouse/human. A TE insert was considered ancient if the pairwise alignment between human and mouse orthologous TE sequences was longer than 100 bp and contained <5% insertions/deletions (stringent definition) or 25% insertions/deletions (relaxed definition). Microarray data for normal (non-cancerous) tissues (73 human and 61 mouse tissues) were used to analyze the lincRNA expression. Log2-normalized median values of expression for each probe set across the tissues were calculated (Managadze et al., 2011). As an alternative method of measuring expression levels, the mouse RNA-seq data for eight tissues (the ENCODE project; modENCODE Consortium) were downloaded from the UCSC genome browser Web site^2^ and pooled together. The RPKM value was calculated for each mouse lincRNA (Managadze et al., 2011). Pairwise evolutionary distances for human–macaque and mouse–rat lincRNA alignments were calculated using the DNADIST program from the PHYLIP package (Felsenstein, 1996), with the Kimura nucleotide substitution model. The lists of lincRNA genes and expression data are available at ftp://ftp.ncbi.nlm.nih.gov/pub/managdav/paper_suppl/TEs_lincRNA/.

One of the problems in the analysis of lincRNAs is that there is little overlap between lincRNA sets produced in different studies (Ulitsky et al., 2011; Chew et al., 2013; Managadze et al., 2013; Schuler et al., 2014). We used human and mouse datasets because these curated lincRNA sets have known evolutionary and gene expression properties (Managadze et al., 2011, 2013). Another reason for this choice is that we sought to analyze lincRNA datasets as different as possible from those used in previous studies (Kapusta et al., 2013; Johnson and Guigo, 2014) and to check how small sample size of human lincRNA set influences results. As shown below, the sample size does not perceptibly affect the conclusions of this study.

## Results

### Transposable elements in human and mouse lincRNA genes

**Figure 1:**
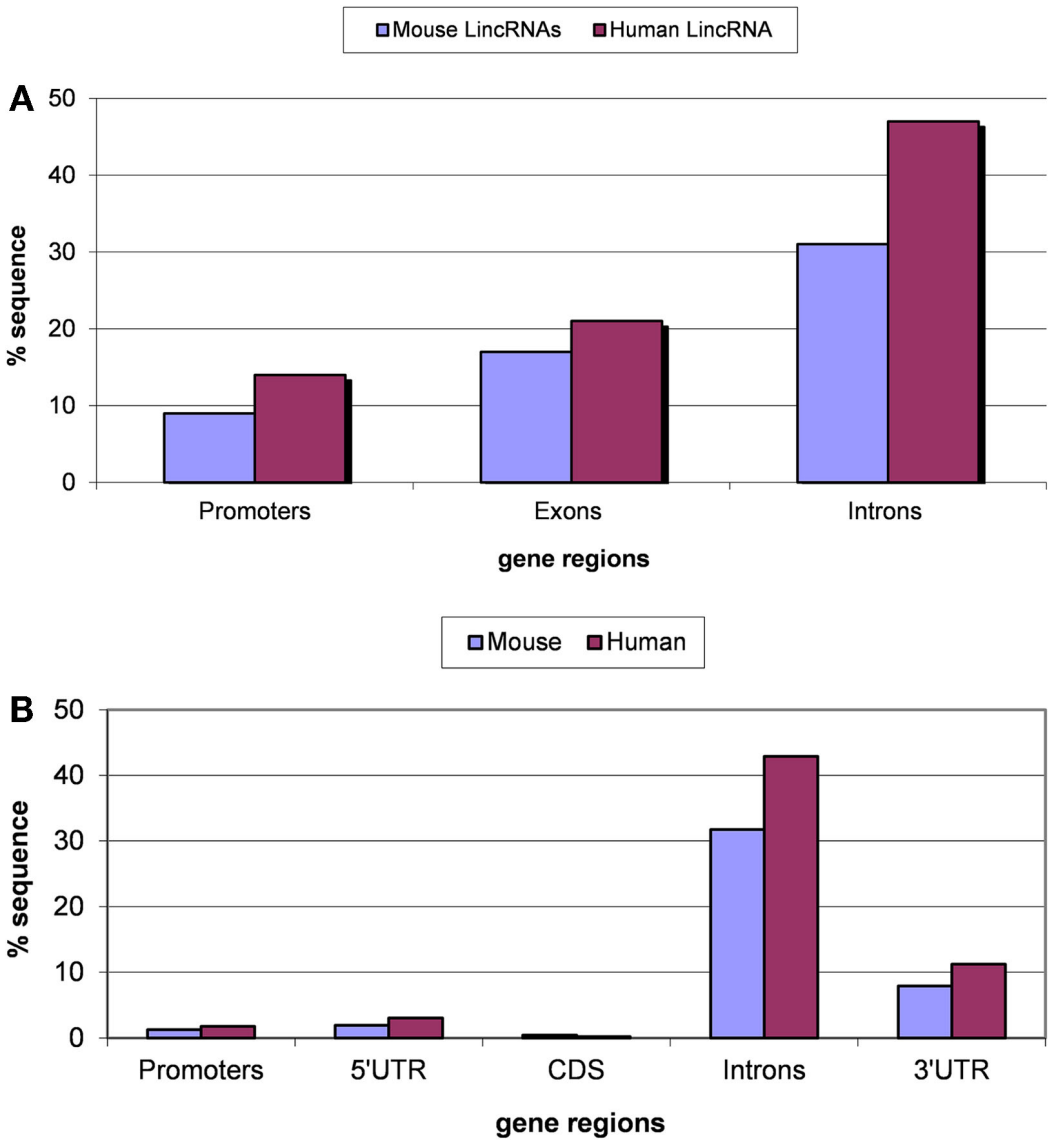
**Fractions (proportions) of lincRNA gene regions (concatenated promoters, exons, and introns) (A) and protein-coding gene regions (B) occupied by TE-derived sequences**. The differences for pairwise comparisons “promoters vs. introns” and “exons vs. introns” are statistically significant for both classes of genes (*P* < 10^−5^ according to the Fisher exact test; the raw counts of nucleotides in TES vs. the raw counts of nucleotides in TE-free regions was used as the input for 2 × 2 contingency tables).

Transposable element-derived sequences (TES for short) comprise at least half of the mammalian genomes, and in particular, are found in most lincRNAs. We identified TES in 69% of the human lincRNAs and 51% of the mouse lincRNAs. These values are somewhat lower than the previously reported 83% of TES in human lincRNAs (Kelley and Rinn, 2012) but nevertheless clearly show the importance of TE for lincRNA evolution. The distribution of TES in 5′ flanking regions (putative core promoter regions), lincRNA exons, and introns is shown in the Figure 1. The lowest fraction of TES was found in the predicted core promoter regions (100 bp upstream regions), and the highest fraction of TES was observed in introns, whereas exonic sequences showed intermediate densities of TES (Figure 1A). This distribution of TES is compatible with the previously described general tendency of TES avoidance in functionally important regions of protein-coding genes (Jordan et al., 2003). In particular, similar to the protein-coding genes, the TES density in extended promoter regions has been found to be significantly greater than that in core promoter regions (Jordan et al., 2003). Notably, the fractions of TES in introns of lincRNAs and protein-coding genes are nearly identical, suggestive of comparable (weak) functional constraints. By contrast, in the exons and the core promoter regions of lincRNA genes, the fractions of TES are substantially and statistically significantly (*P* < 10^−5^ according to the Fisher exact test) higher than in the respective regions of protein-coding genes (compare Figures 1A,B). These findings are consistent with the results of a previous study that employed different datasets of lincRNA genes (Kapusta et al., 2013), indicating that the distribution of TES in lincRNA genes is a robust feature. A more detailed analysis of the distribution of TES across lincRNAs is shown in Figure S1 in Supplementary Material. The most prominent feature of this distribution is the high fraction of lincRNAs with a low TES content: in 66% of human lincRNAs and 78% of mouse lincRNAs, the fraction of TES is <20% (Figure S1 in Supplementary Material).

**Figure 2:**
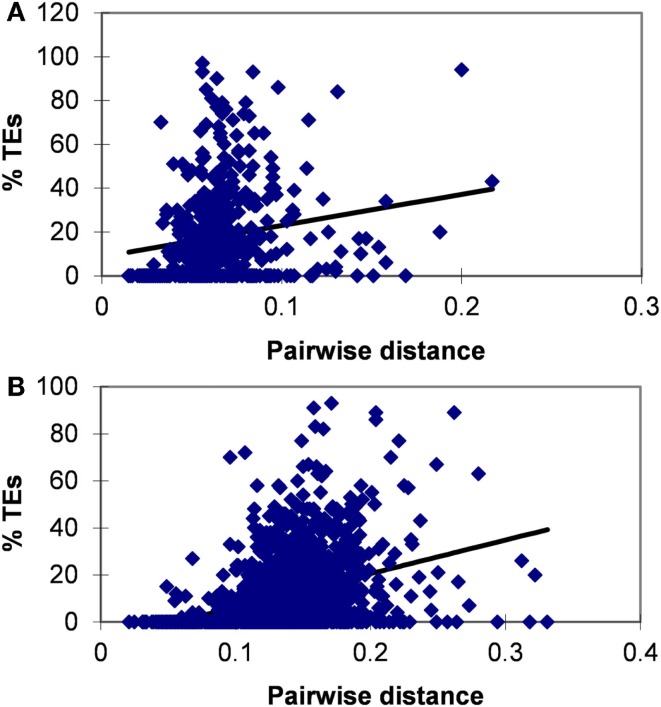
**Correlation between the fraction (proportion) of TEs in concatenated exons and evolutionary rate for human (A) and mouse (B) lincRNAs**. Pearson correlation coefficient is 0.183 for human and 0.337 for mouse (*P* < 10^−5^ for both comparisons).

The avoidance of TES in lincRNAs is consistent with purifying selection, which is an important feature of lincRNA evolution (Ponjavic et al., 2007; Managadze et al., 2011). The significant positive correlation between the evolutionary rate and the content of TES was observed for both human and mouse lincRNA sets (for human and mouse, respectively, the Pearson correlation coefficient are 0.183 and 0.337, *P* < 10^−5^ for both comparisons) (Figure 2). We also tested the correlation between the expression level and the content of TES (Figure S2 in Supplementary Material). In many independent previous studies, it has been shown that protein-coding genes that are highly expressed in many tissues typically evolve slower than genes with lower expression level and breadth (Duret and Mouchiroud, 2000; Krylov et al., 2003; Drummond and Wilke, 2008), and a similar dependence has been observed for lincRNA genes (Managadze et al., 2011). Consistent with these observations, here we found a significant negative correlation between the content of TES and the expression level of lincRNAs (Figure S2 in Supplementary Material; for mouse RNA-seq data, Pearson correlation coefficient is −0.158, *P* < 10^−5^; for mouse microarray data, Pearson correlation coefficient is −0.07, *P* < 10^−5^; for human microarray data, Pearson correlation coefficient is −0.253, *P* < 10^−5^).

### Different classes of transposable elements in lincRNA genes

**Figure 3:**
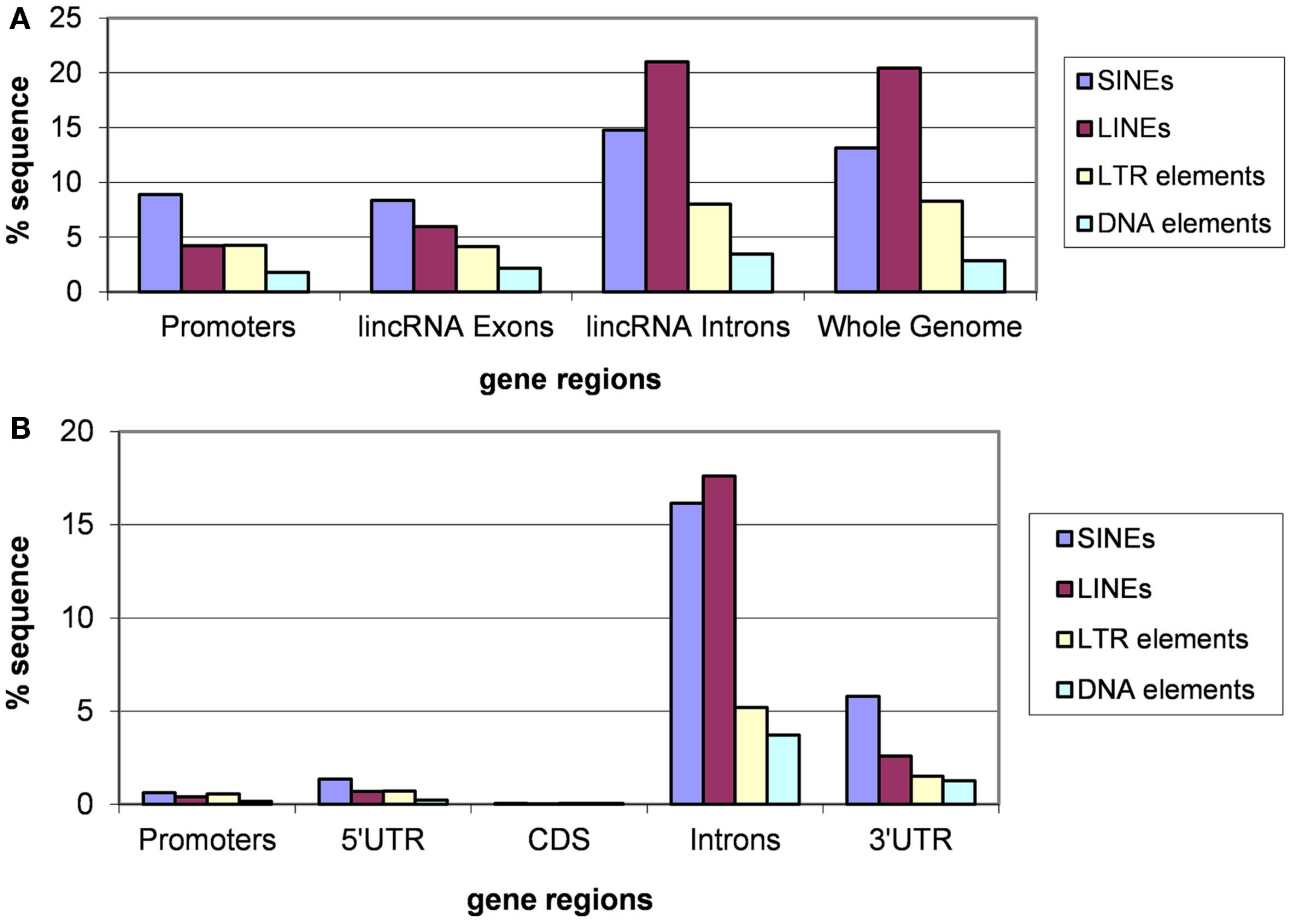
**Fractions (proportions) of human lincRNA gene regions (concatenated promoters, exons, and introns) and the whole genome sequence (A) and protein-coding gene regions (B) occupied by sequences derived from different types of TEs**. Differences for pairwise comparisons “SINEs vs. LINEs” and “LTRs vs. LINEs” are statistically significant for both classes of genes (*P* < 10^−5^ according to the Fisher exact test; the raw counts of nucleotides in TES vs. the raw counts of nucleotides in TE-free regions was used as the input for 2 × 2 contingency tables).

**Figure 4:**
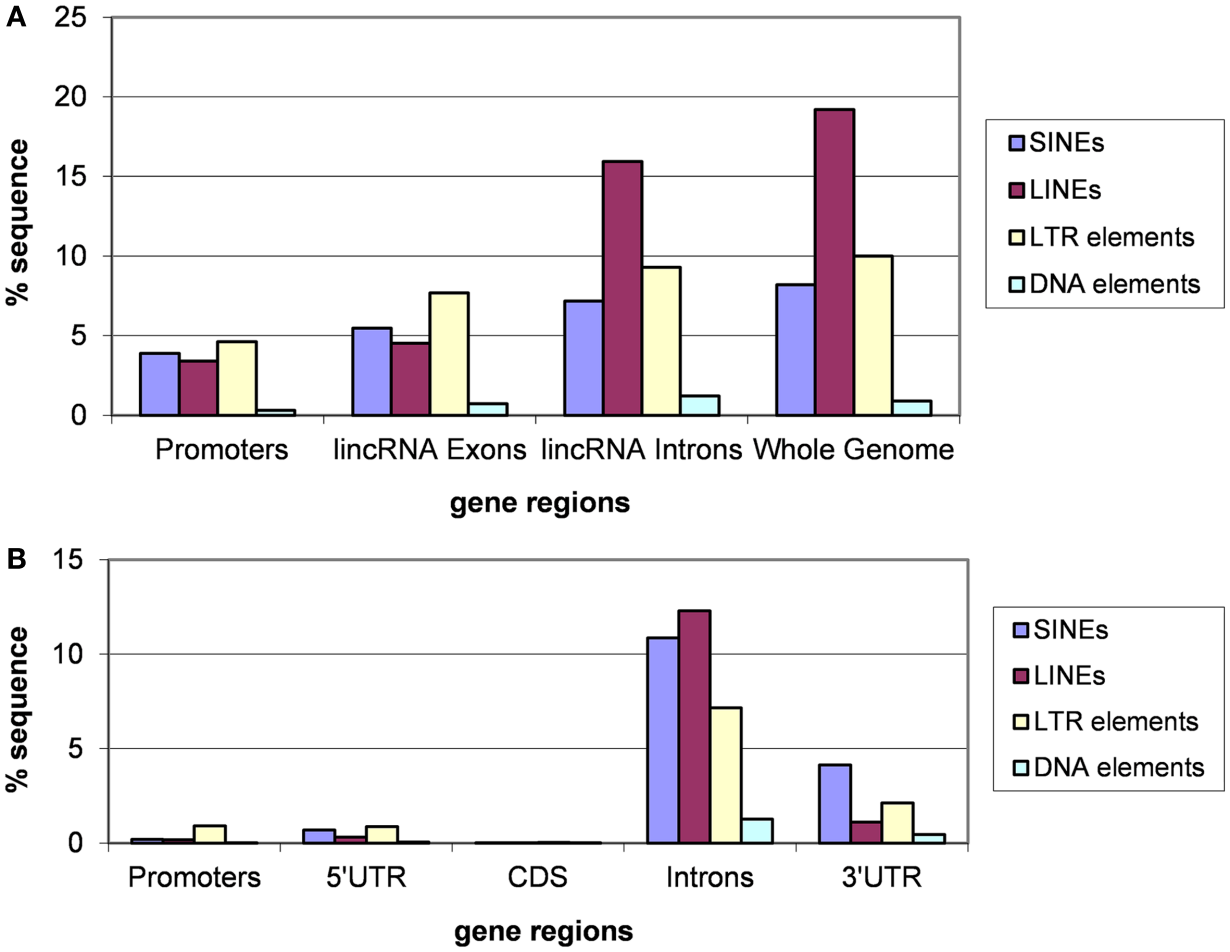
**Fractions (proportions) of mouse lincRNA gene regions (concatenated promoters, exons, and introns) and the whole genome sequence [the data for the whole genome sequence were from Waterston et al. (2002)] (A) and protein-coding gene regions (B) occupied by sequences derived from different types of TEs**. Differences for pairwise comparisons “SINEs vs. LINEs” and “LTRs vs. LINEs” are statistically significant for both classes of genes (*P* < 10^−5^ according to the Fisher exact test; the raw counts of nucleotides in TES vs. the raw counts of nucleotides in TE-free regions was used as the input for 2 × 2 contingency tables).

Analysis of different classes of TEs indicates that the fractions of each class are similar for introns of lincRNA and protein-coding genes and whole genomes (Figures 3 and 4). In each case, the fraction of LINEs is substantially greater than those of SINEs and LTR elements (Figures 3A and 4A). However, there is a significant suppression of LINEs in exonic and promoter regions, in both human and mouse (Figures 3A and 4A). This effect cannot be explained by fluctuations of the base composition in different gene regions because there are no significant compositional differences between exons, introns, and promoter regions for human and mouse lincRNA genes (results not shown). The same trend was observed for different lincRNA sets (Kapusta et al., 2013) suggesting that re-distribution of TEs is a general property of mammalian lincRNA genes. Furthermore, similar tendency is observed in promoter sequences of protein-coding genes (Figures 3B and 4B), the overall lower abundance of TEs notwithstanding. Conceivably, when the smaller SINEs are inserted into functionally important parts of genes, they typically exert a milder deleterious effect than the larger LINEs and LTR elements and accordingly, are more often fixed in the course of evolution.

### Higher frequency of ancient transposable element-Derived sequences in promoters and exons compared to introns

**Figure 5:**
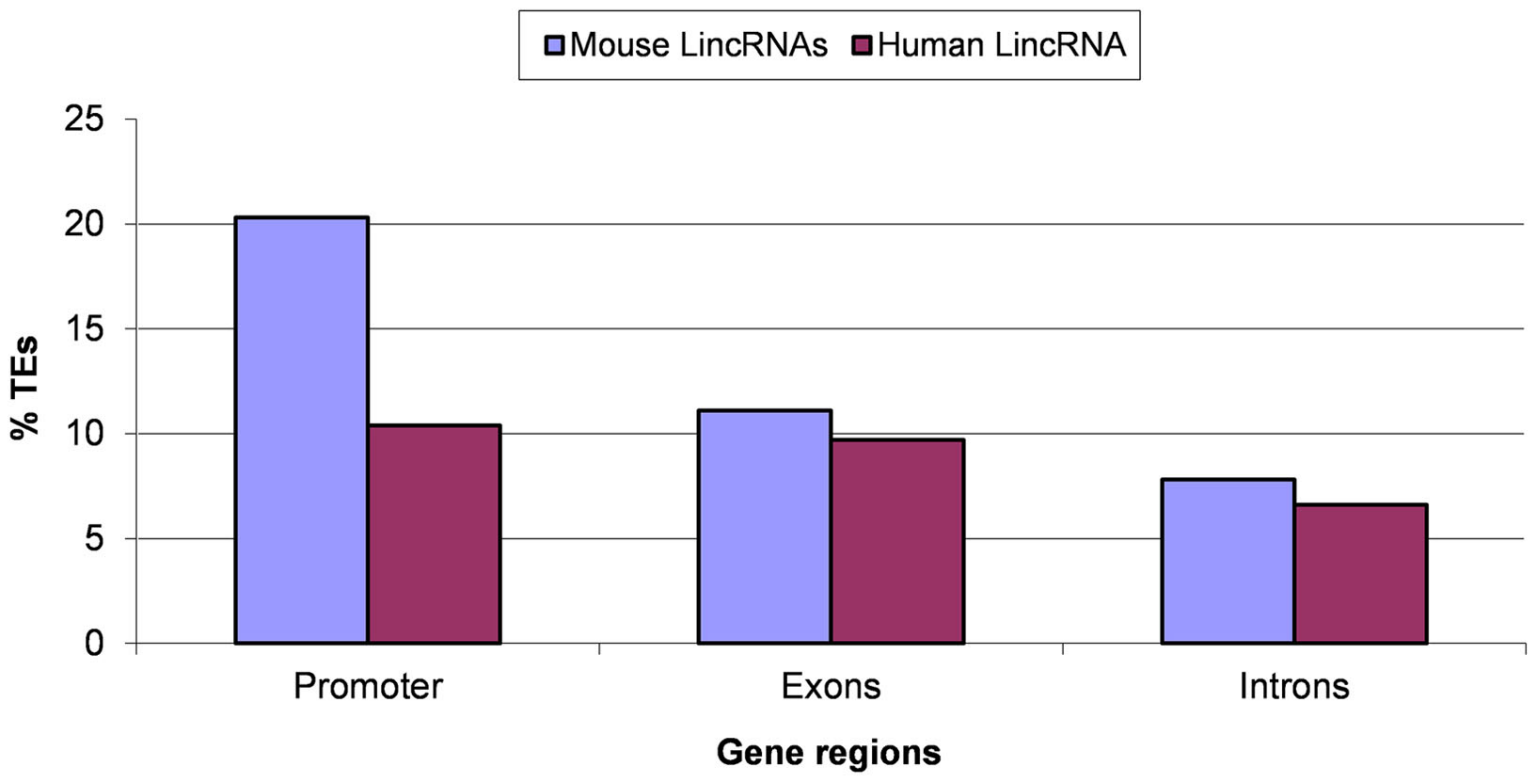
**Ancient transposable elements (TEs) in putative promoter regions, exons, and introns of lincRNA genes**. A TE was considered ancient if the alignment between human–mouse orthologous TE sequences was longer than 100 bp and contained <5% insertions/deletions (the stringent threshold). “% TEs” stands for the fraction (proportion) of ancient TEs. Results for the relaxed threshold (the alignment between human–mouse orthologous TE sequences was longer than 100 bp and contained no more than 25% insertions/deletions) are shown in the Table S2 in Supplementary Material. Differences between pairwise comparisons “promoters vs. introns” and “exons vs. introns” are statistically significant (*P* < 10^−5^ according to the Fisher exact test; the raw counts of ancient TES vs. the raw counts of lineage-specific TES was used as the input for 2 × 2 contingency tables).

Evolutionary conservation of TEs is likely to reflect molecular domestication of the respective elements (Jordan et al., 2003; Feschotte, 2008; Jurka, 2008; Bourque, 2009; Sinzelle et al., 2009). We analyzed the fraction of ancient mobile elements in different regions of lincRNA genes (Figure 5). A significantly higher abundance of ancient TEs (*P* < 10^−5^ according to the Fisher exact test) was detected in exons and especially in promoter regions compared to introns (Figure 5). This finding is consistent with the hypothesis that TEs, in some cases, may perform novel functions in the host organisms (Makalowski, 2000; Jordan et al., 2003). The excess of ancient TEs was more pronounced in human compared to mouse lincRNA genes (Figure 5), possibly reflecting differences in evolutionary processes in rodents and primates although a bias caused by technical problems with the detection of 5′-ends of human lincRNA sequences cannot be ruled out (Kutter et al., 2012). We searched the putative promoter regions of lincRNA genes for the presence of TATA boxes and found a substantially elevated frequency of TATA-like sequences in the region −25 to −35 (Figure S3 in Supplementary Material). Given that a similar distribution is observed in many well-annotated human protein-coding genes (Yang et al., 2007), these observations suggest acceptable accuracy of 5′-end identification in lincRNA genes. The fractions of TATA-containing promoters are similar for protein-coding genes (10–25%) (Yang et al., 2007; Anish et al., 2009) and the analyzed sets of lincRNA genes (19–30%; Table S1 in Supplementary Material). The higher frequency of ancient TEs in promoters and exons compared to introns (Figure 5; Table S2 in Supplementary Material) suggests that many lincRNA genes emerged before the split of primates and rodents, and that TEs contributed to the origin of these ancient lincRNAs. This finding is consistent with recent observations that 60–70% of the lincRNAs genes are conserved between human and mouse (Kutter et al., 2012; Managadze et al., 2013), and with the observed higher conservation of lincRNA promoter regions compared to exons (Elisaphenko et al., 2008; Kapusta et al., 2013; Johnson and Guigo, 2014).

## Discussion

The staggering evolutionary success of TEs in eukaryotes is often attributed to their ability to out replicate the host genomes in which they reside, as opposed to any selective advantage that they might provide to their hosts. Indeed, it has been shown that TEs can spread within and among genomes even in the face of a selective cost to the host (Hickey, 1982). Hence, the selfish DNA concept of TEs focuses on the parasitic nature of these elements and emphasizes the deleterious effects of transposition as well as the negligible evolutionary benefit that TEs provide to their hosts (Orgel et al., 1980; Gould and Vrba, 1982). However, the sheer abundance of TEs in the genome, as well as the variety of mutation effects induced by their mobility, suggest that they might, in some cases, be exapted (Gould and Vrba, 1982) or domesticated (Miller et al., 1999), to serve the evolutionary interests of the host (Makalowski, 2000; Jordan et al., 2003). Indeed, multiple lines of evidence indicate that the presence of TEs can result in host adaptation by shaping and reshaping the genome in many different ways (Smit, 1996; Makalowski, 2000; Rogozin et al., 2000; Kidwell and Lisch, 2001; Deininger and Batzer, 2002; Jordan et al., 2003).

The TEs comprise at least half of the mammalian genomes, and in particular, are found in most lincRNAs [this study and Kelley and Rinn (2012)]. Here, we demonstrate that TEs substantially contribute to the evolution of lincRNAs and their promoter regions. Although the densities of TES in these regions are much lower than those in introns, ostensibly, due to the purifying selection that affects functional regions, the contributions of TEs to the evolution of these regions is substantially greater than in the respective regions of protein-coding genes. The higher density of TES in the exons of lincRNAs compared to protein-coding exons appears to reflect the much lower level of functional constraint characteristic of the former (Ponjavic et al., 2007; Managadze et al., 2011). The promoters of lincRNA appear to similarly enjoy greater plasticity and flexibility compared to the promoters of protein-coding genes.

Thus, TE insertion is an important factor that affects lincRNA evolution and biological function. An analysis of TEs in human lincRNAs revealed that the TES composition in lincRNA genes significantly differs from genomic averages: LINEs and SINEs are depleted whereas LTR retrotransposons are enriched (Kelley and Rinn, 2012). The TES occur in biased positions and orientations at lincRNA transcription start sites suggesting a functional role in lincRNA transcriptional regulation (Kelley and Rinn, 2012). In many cases, lincRNAs devoid of TES are expressed at higher levels than lincRNAs containing TES in all tested tissues and cell lines (Kelley and Rinn, 2012). Thus, it has been suggested that TES divide lincRNAs into classes and have contributed to lincRNA evolution and function by conferring tissue-specific expression from extant transcriptional regulatory signals (Kelley and Rinn, 2012). Here, we add another facet to these observations by showing that the promoter regions of lincRNAs are specifically enriched for ancient TES. This finding indicates that not only have many lincRNA genes evolved before the radiation of primates and rodents but also that at least some features of their regulation were already established at that time through TE insertion.

The possibility that some lincRNA genes encode short peptides that are translated, perhaps in a tissue-specific manner, is the subject of an ongoing debate (Brosius and Tiedge, 2004; Mattick and Makunin, 2006; Dinger et al., 2008; Makalowska et al., 2010; Carvunis et al., 2012; Chew et al., 2013). It is extremely hard to rule out such a role for a fraction of purported lincRNAs. A recent peptidomics study demonstrated that most annotated lincRNAs do not generate stable protein (peptide) products (Banfai et al., 2012). Furthermore, ribosomal profiling of lincRNAs suggests that ribosomal engagement with lincRNAs is likely to be regulatory (Chew et al., 2013). The presence of ORFs in the analyzed lincRNA data sets had been analyzed before using different approaches (Managadze et al., 2011, 2013). Importantly, removal of ORF-containing lincRNAs did not affect the conclusions of both studies (Managadze et al., 2011, 2013). The much higher abundance of TES in lincRNA compared to 5′UTR and protein-coding regions of mRNAs is consistent with the low frequency of protein-coding regions in the analyzed data sets.

It has been proposed that lncRNAs are organized into combinations of discrete functional domains, but the nature of these domains and their identification remain elusive (Guttman and Rinn, 2012). Insertion of TEs and exaptation of TES could represent an important route of evolution of the domain structure of lncRNAs. More specifically, Johnson and Guigo (2014) have proposed that exonic TES comprise functional domains of lncRNAs that they dubbed repeat insertion domains of LncRNAs (RIDLs). A growing number of RIDLs have been experimentally identified whereby lncRNA TES function as RNA-, DNA-, and protein-binding domains/motifs (Elisaphenko et al., 2008; Kelley and Rinn, 2012; Grote and Herrmann, 2013; Holdt et al., 2013; Johnson and Guigo, 2014). These examples are likely to reflect a more general phenomenon of exaptation and/or domestication during lncRNA evolution whereby TES are employed as DNA-, RNA-, and protein-binding domain/motifs (Johnson and Guigo, 2014). The RIDL hypothesis has the potential to explain how functional evolution can keep pace with the fast evolution observed in many lncRNA genes (Johnson and Guigo, 2014). The findings on the distribution of TES across different regions of lincRNA genes, the higher occurrence of TES in lincRNA promoters and exons compared to introns, and significant correlations between the content of TES and evolutionary rate presented here appear to be compatible with the RIDL hypothesis. More specifically, even if a substantial fraction of TES are not fixed in lincRNA exons and promoter regions, those TES that are fixed tend to persist in the genome longer than intronic TES. Moreover, given the near ubiquity of recognizable TES in lincRNA genes, TE mapping can be a useful approach for characterization of lincRNAs and possibly even prediction of their functions. The correlations between the content of TES and various features of lincRNA genes described here could be useful for the characterization of lincRNA functions.

## Conclusion

The results of the present analysis, along with several previous studies, indicate that TEs have contributed to the evolution of many if not most mammalian lincRNAs. Whereas the density of TES in the introns of lincRNA genes is about the same as in introns of protein-coding genes exons, and promoters of lincRNAs are markedly enriched in TES compared to the counterparts in protein-coding genes. This high prevalence of TES reflects the relatively weak evolutionary constraints on lincRNA genes and itself appears to contribute to the plasticity and functional diversification of lincRNAs. Furthermore, the distribution of TE types in the functional regions of lincRNA genes significantly differs from that in introns (or whole genomes), conceivably, because the smaller SINEs that encode no proteins are more suitable for exaptation than the larger, protein-coding LINEs. The prodigious exaptation of TE could account, at least in part, for the functionality of many lincRNAs despite their rapid evolution.

## Conflict of Interest Statement

The authors declare that the research was conducted in the absence of any commercial or financial relationships that could be construed as a potential conflict of interest.

## Supplementary Material

The Supplementary Material for this article can be found online at http://www.frontiersin.org/article/10.3389/fbioe.2015.00071/abstract

## Footnotes

^1^http://www.repeatmasker.org

^2^http://hgdownload.cse.ucsc.edu/goldenPath/mm9/encodeDCC/wgEncodeLicrRnaSeq/
