# Supplementary material for: Transposable Element Insertions in Long Intergenic Non-Coding RNA Genes
Source: Front Bioeng Biotechnol. 2015 Jun 9;3:71. doi: 10.3389/fbioe.2015.00071 (PMC4460805; doi:10.3389/fbioe.2015.00071)
Supplement: Supplementary file 1 [file datasheet_1.pdf]

Supplementary Materials

Transposable element insertions in long intergenic non-coding RNA genes

Supplementary Figure 1. Fraction of human (A) and mouse (B) lincRNAs (concatenated exons) occupied by TE-derived sequences.

(A)

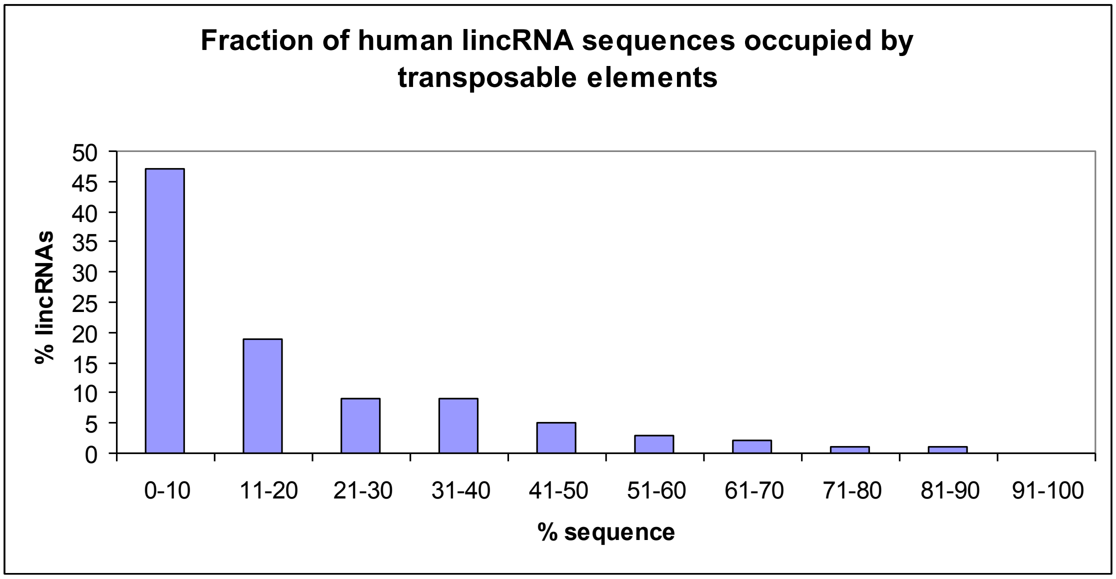

(B)

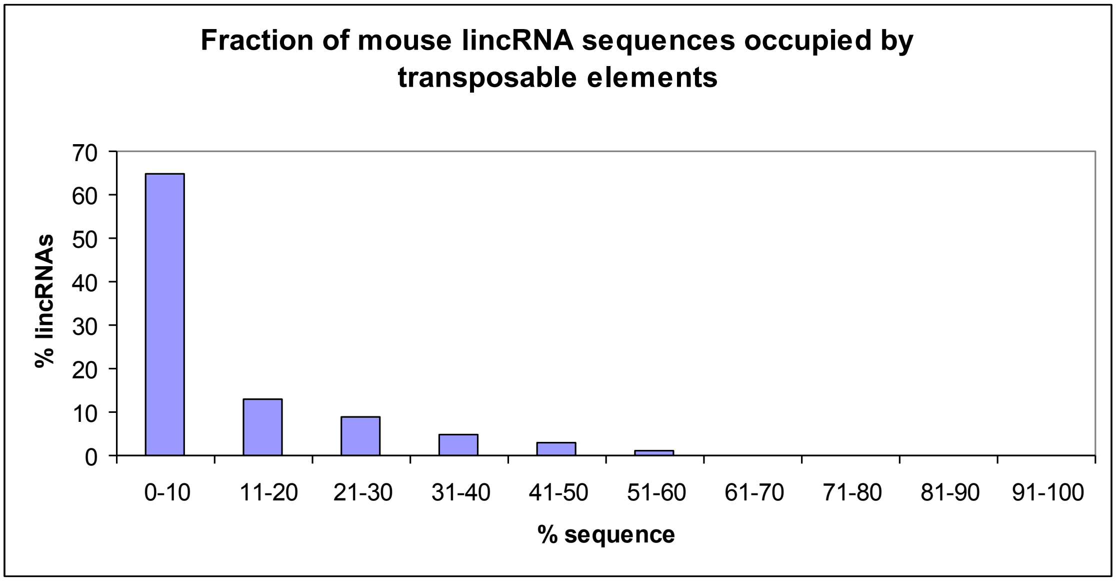

Supplementary Figure S2. Correlation between the expression level and the fraction (proportion) of TEs in concatenated exons for (A) mouse lincRNAs based on RNAseq data, (B) for mouse lincRNAs based on microarray data, and (C) human lincRNAs based on microarray data.

(A)

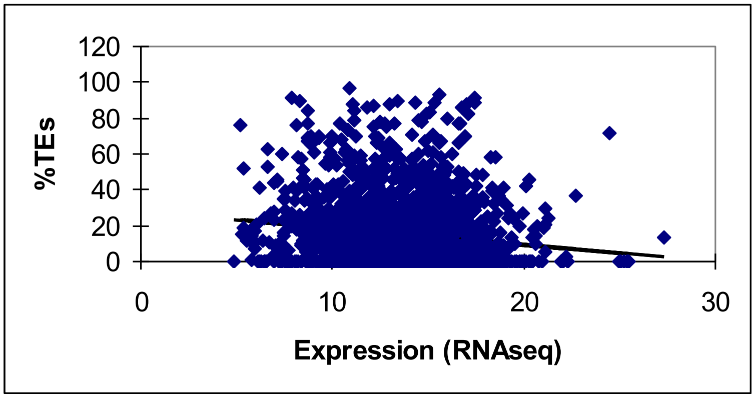

(B)

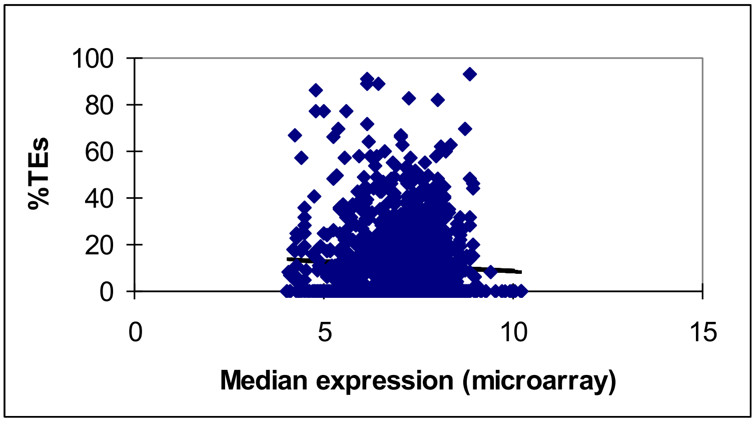

(C)

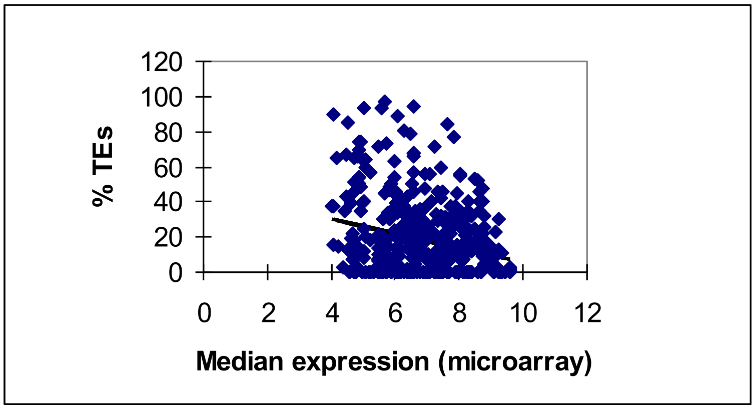

Supplementary Figure S3. Search for TATA motifs in human and mouse lincRNA promoter regions (Upstream 100 nt). A) TATA-motifs used in this study. The six motifs in IUPAC notation and their names in parenthesis are as follows: TATAAA (TATA), TATAWAWR (TATA-8), TATA[An][An] (TATA, 1 mismatch allowed in positions with "n"), TATA[Wn][An][Wn][Rn] (TATA-8, 1 mismatch allowed in positions with "n"), ATAWTA (TATA Shuffled) and TWAARATW (TATA-8 Shuffled). Only one mismatch is allowed in the variable positions (denoted by "n") for 1-mismatch motifs. Shuffled motifs are generated by randomly shuffling the TATA and TATA-8 motifs. B) Distribution of TATA box in 589 Human lincRNA putative promoters (upstream 100 nt). (C) Distribution of TATA box in 2,390 Mouse lincRNA putative promoters (upstream 100 nt).

(A)

TATA

TATA with 1 mismatch

Shuffled TATA

TATA8

TATA8 with 1 mismatch

Shuffled TATA8

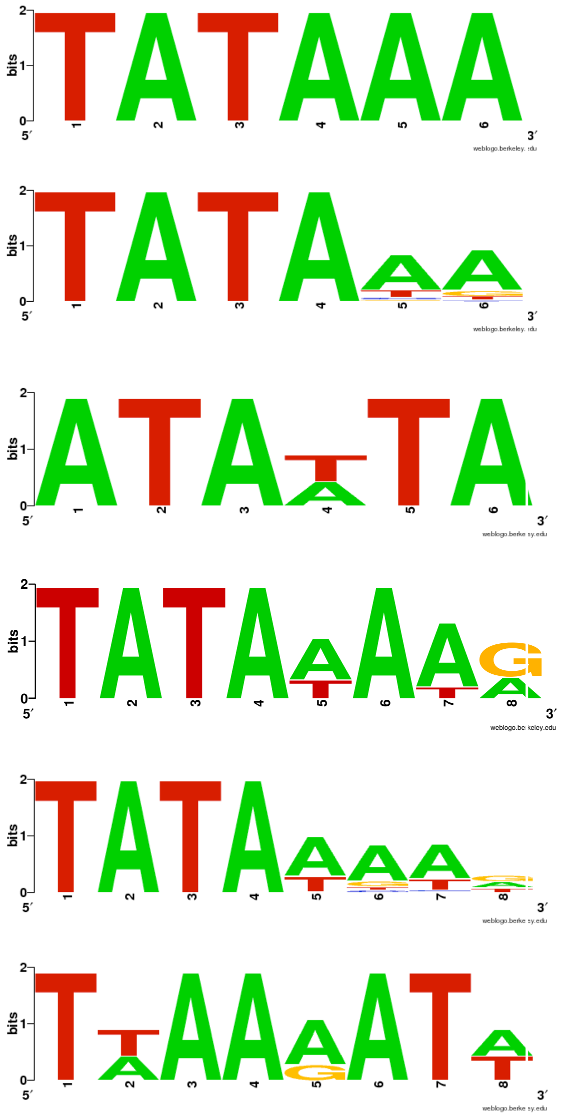

(B)

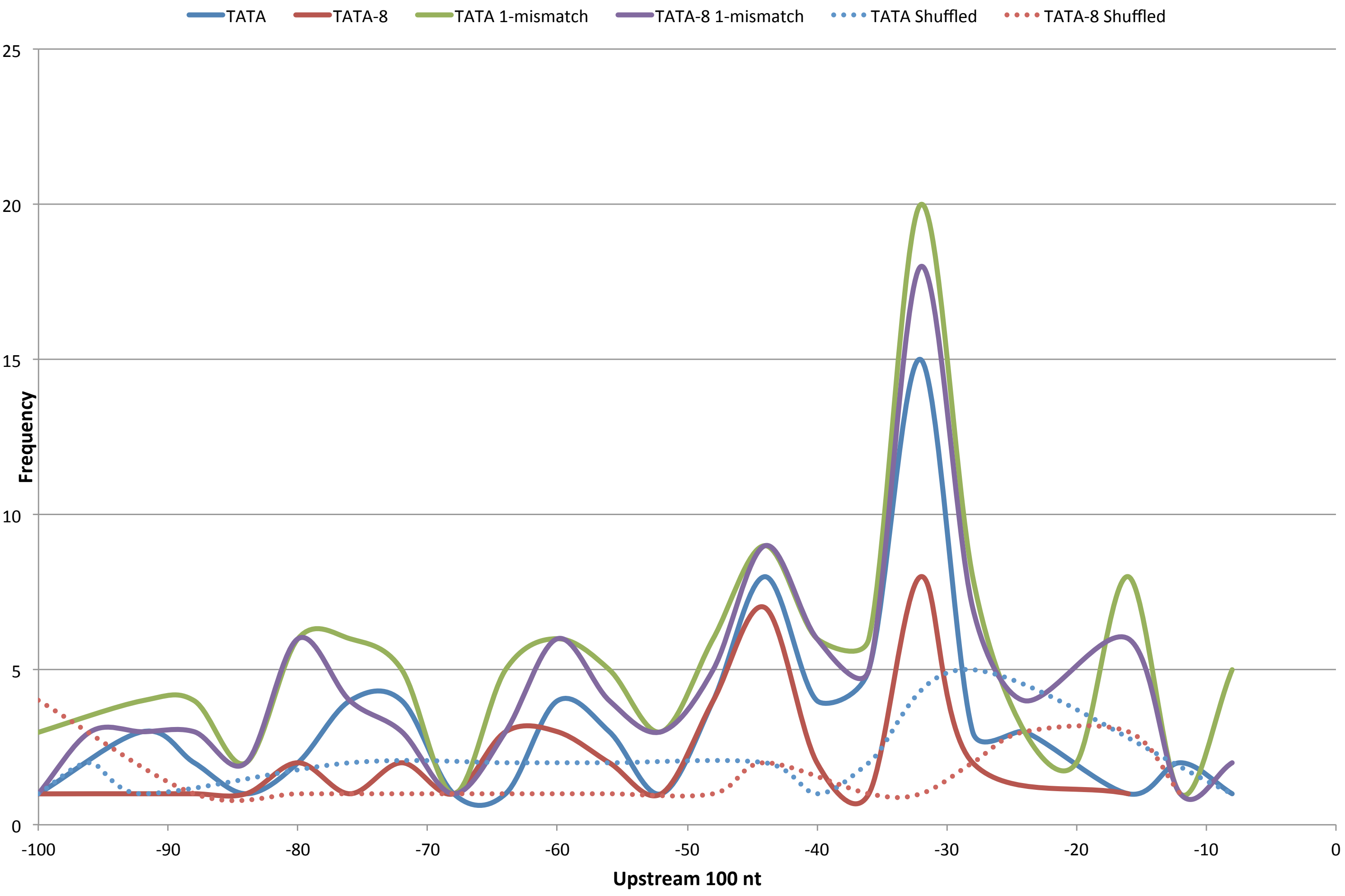
TATA
TATA-8
TATA 1-mismatch
TATA-8 1-mismatch
TATA Shuffled
TATA-8 Shuffled
Frequency
0
5
10
15
20
25
-100
-90
-80
-70
-60
-50
-40
-30
-20
-10
0
Upstream 100 nt

(C)

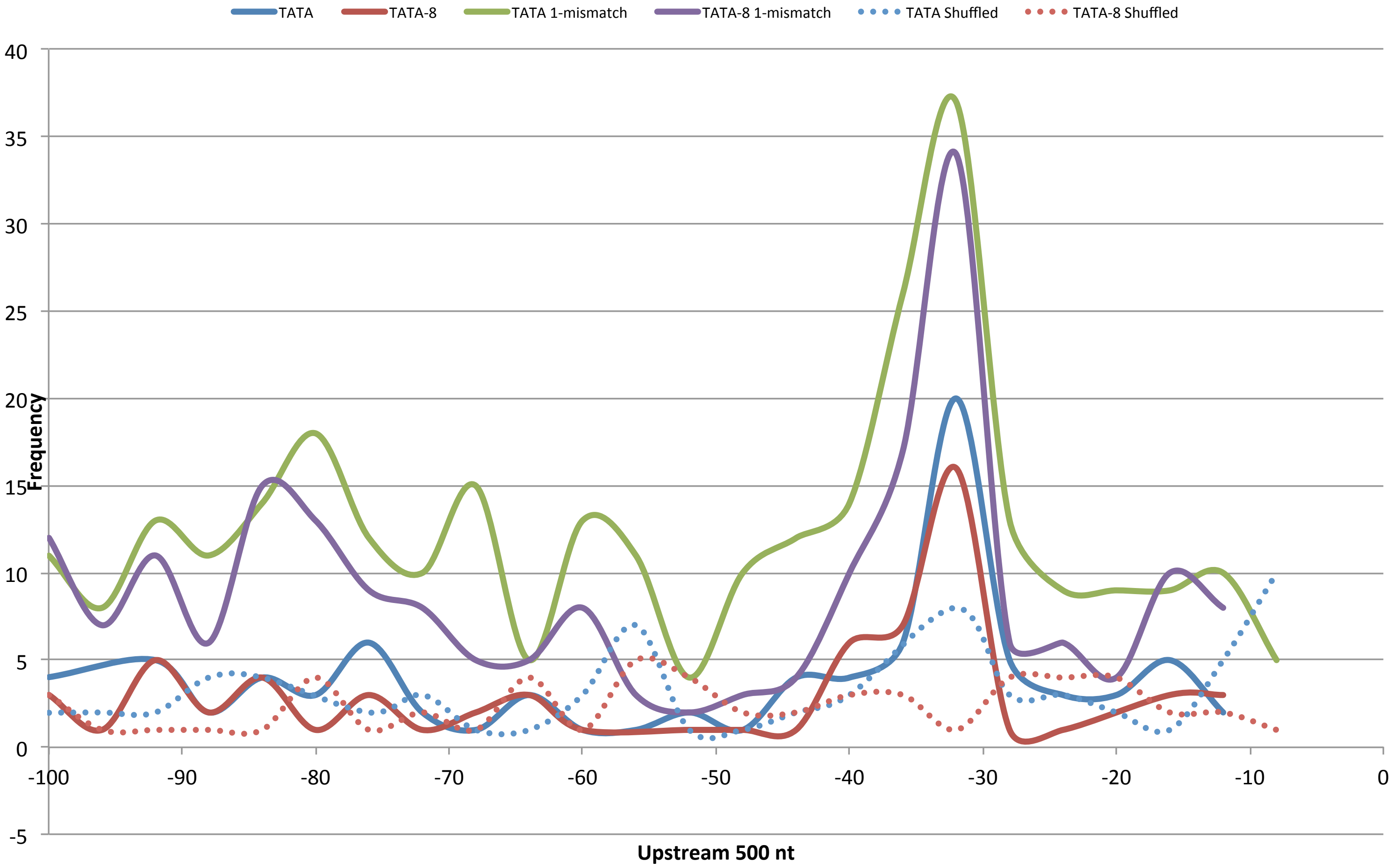

TATA
TATA-8
TATA 1-mismatch
TATA-8 1-mismatch
TATA Shuffled
TATA-8 Shuffled
Frequency
40
35
30
25
20
15
10
5
0
-5
-100
-90
-80
-70
-60
-50
-40
-30
-20
-10
0
Upstream 500 nt

Supplementary Table S1. The fraction of TATA-containing promoters.

TATA motif	Species	lincRNA genes	Protein-coding genes
TATA with 1 mismatch	Human	30%	12%
	Mouse	19%	15%

The fraction was calculated using the "TATA with 1 mismatch" model (see the Supplementary Figure S1 for details). Differences for pairwise comparisons "lincRNA genes vs. protein-coding genes" are statistically significant ($P < 10^{-5}$ according to the Fisher exact test; the raw counts of ancient TATA-containing promoters vs. the raw counts of TATA-less promoters was used as the input for 2 x 2 contingency tables) although we cannot exclude that this is caused by biases in local oligonucleotide composition.

Supplementary Table S2. Ancient transposable elements (TEs) in putative promoter regions, exons and introns of lincRNA genes.

Species	Functional region	% TEs that are ancient (stringent)	% TEs that are ancient (relaxed)
Mouse	Promoters	20.3	27.2
	Exons	11.1	16.3
	Introns	7.8	11.9
Human	Promoters	10.4	27.7
	Exons	9.7	25.6
	Introns	6.6	20.4

A TE was considered ancient if the alignment between human/mouse orthologous TE sequences was longer 100 bp and contains less than 5% insertions/deletions (the stringent threshold) or less than 25% insertions/deletions (the relaxed threshold). Differences for pairwise comparisons "promoters vs. introns" and "exons vs. introns" are statistically significant ($P < 10^{-5}$ according to the Fisher exact test; the raw counts of ancient TES vs. the raw counts of lineage-specific TES was used as the input for 2 x 2 contingency tables).
